# Soft tissue reconstruction techniques for irreparable anterosuperior rotator cuff tears: A systematic review of clinical outcomes

**DOI:** 10.1177/17585732261431826

**Published:** 2026-03-18

**Authors:** Justin Gilbert, Marc Daniel Bouchard, Colin Kruse, Prushoth Vivekanantha, Darius Luke Lameire, Danielle Dagher, Moin Khan

**Affiliations:** 1 Michael G. DeGroote School of Medicine, 12362 McMaster University, Hamilton, ON, Canada; 2 574408 Division of Orthopaedic Surgery, 3710 McMaster University, Hamilton, ON, Canada; 3 Department of Health Research Methods, Evidence, and Impact, 152642 McMaster University, Hamilton, ON, Canada; 4 Division of Orthopaedic Surgery, University of Toronto, Hamilton, ON, Canada

**Keywords:** Irreparable rotator cuff tear, anterosuperior rotator cuff, tendon transfer, pectoralis major transfer, latissimus dorsi transfer, shoulder reconstruction

## Abstract

**Background:**

Irreparable anterosuperior (AS) rotator cuff tears involving the subscapularis and supraspinatus cause significant shoulder dysfunction, instability, and pain. Surgical options such as pectoralis major (PM), pectoralis minor (Pm), and latissimus dorsi (LD) transfers aim to restore shoulder biomechanics, but the optimal technique remains uncertain. This systematic review evaluated clinical outcomes of soft tissue reconstruction for irreparable AS tears.

**Methods:**

Following PRISMA guidelines, Ovid MEDLINE, Embase, Emcare, and PubMed were searched. Eligible studies included randomized trials, cohort studies, and case series (≥10 patients). Methodological quality was assessed using the MINORS criteria.

**Results:**

Eight studies (350 patients) evaluating PM, Pm, LD, and combined latissimus dorsi–teres major (LDTM) transfers were included. Pm and LD transfers demonstrated the lowest failure (1.0%, 0%) and complication rates (0%, 5.6%). PM and LDTM transfers showed higher failure (12.2%) and complication rates (13.5%, 1.6%). All techniques improved patient-reported outcomes. Improvements in range of motion were most consistent in shoulder elevation, while rotational gains were smaller and variably reported.

**Conclusion:**

Soft tissue reconstruction for irreparable AS rotator cuff tears is associated with improved clinical outcomes. Pm and LD transfers appear to offer a favorable balance of functional improvement and safety, although study heterogeneity limits definitive comparison.

## Introduction

The anterosuperior (AS) rotator cuff, consisting of the subscapularis and supraspinatus muscles, plays a critical role in maintaining glenohumeral stability and facilitating a broad range of shoulder motions.^1^ These muscles work together to maintain the humeral head within the glenoid during arm movement, and are essential for anterior shoulder stability.^2^ Tendon fibers of the upper subscapularis fuse with the anterior subscapularis tendon, forming the anterior cable which particularly stabilizes the humeral head during arm elevation.^3^ Tears of the supraspinatus tendon usually extend posteriorly, involving the infraspinatus tendon.^4^ Anterior involvement is much less common, and has been investigated less thoroughly than posterosuperior and isolated tears of the rotator cuff.^4,5^ However, when these tears extend anteriorly to affect the subscapularis, the anterior cable structure is disrupted resulting in marked destabilization of the humerus.^3,4^ Additionally, these AS injuries frequently compromise the long head of the biceps due to inflammation and distension, further evoking pain and disrupting arm function.^3,6^

When tears in AS rotator cuff become irreparable, defined as tears in which primary repair is not feasible due to chronic tendon retraction, poor tissue quality, advanced fatty infiltration, and/or muscle atrophy despite attempted mobilization, the resulting dysfunction can severely impair shoulder mobility, strength, and stability.^7–9^ Persistent pain and significant limitation in performing daily activities is commonly experienced.^1–5^ Timely and effective management of irreparable tears is crucial to preserve joint function and limit the progression of cuff tear arthropathy.^6–8^ Given the loss of structural integrity of these irreparable tears, significant grafting of tissues is required to restore some function of the shoulder.

Various soft tissue reconstruction techniques have been proposed to treat irreparable AS rotator cuff tears, each with unique biomechanical rationales and indications. Tendon transfers with pectoralis major (PM), pectoralis minor (Pm), latissimus dorsi (LD), and teres minor (TM) have all been investigated.^7,8^ Typically, LD transfers have been implicated for posterosuperior tears while the PM tendon has been favored for AS tears, given the relative anatomic positions of these tendons.^7,9^ With its anterior location and relatively less invasive procedure compared to PM, pectoralis minor transfers have also been suggested for AS tears.^7–9^ Reverse total shoulder arthroplasty (rTSA) remains reserved as a salvage option for failed tendon transfers and retears, or for elderly patients with glenohumeral arthritis.^7–9^ Although these techniques can restore shoulder force coupling and enhance stability, the optimal management strategy for irreparable AS rotator cuff tears remains controversial. While posterosuperior tears are the most common and extensively studied pattern, irreparable AS tears are comparatively underrepresented in the literature.^4,5^ They pose unique biomechanical challenges yet there remains a relative paucity of high-quality evidence guiding their treatment compared to the wealth of data available for posterosuperior tears.^5^ By synthesizing and analyzing the existing literature, this study aimed to evaluate the relative effectiveness of various soft-tissue reconstructive options for irreparable AS tears while also examining the scope and quality of available evidence to identify current gaps in knowledge and guide future research.

## Methods

### Search strategy

This systematic review was conducted in accordance with the Preferred Reporting Items for Systematic Reviews and Meta-Analyses (PRISMA) guidelines.^10^ A comprehensive search of Ovid MEDLINE, Embase, and Emcare databases was performed from inception to April 6, 2025, with PubMed searched from inception to January 29, 2026. The search strategy combined terms related to AS rotator cuff anatomy (“anterior,” “superior,” “anterosuperior”), rotator cuff pathology (“rotator cuff,” “shoulder,” “tear,” “rupture,” “instability,” “injury”), and irreparability (“irreparable,” “unfixable,” “irrecoverable,” “irreversible,” “unrepairable,” “incurable,” “unmendable”). Boolean operators (AND, OR) were used to systematically combine concepts. No language restrictions were applied. The complete search strategies for each database are provided in Supplementary Table 1. Reference lists of all included articles, as well as relevant prior systematic reviews, were manually screened to ensure comprehensive capture of eligible studies.

### Eligibility criteria and study selection

Studies were eligible for inclusion if they investigated the surgical management of irreparable AS rotator cuff tears and reported clinical outcomes following tendon transfer or reconstructive techniques. Eligible study designs included randomized controlled trials (RCTs), prospective or retrospective cohort studies, and case series with a minimum of 10 patients. Included studies were required to report at least one clinical outcome measure, such as functional scores, range of motion, pain relief, complication rates, or structural integrity. Studies were excluded if they focused solely on posterosuperior tears, arthroplasty procedures, or non-operative management, or if they were case reports, technical notes without clinical outcomes, or cadaveric biomechanical studies. Articles not available in English were excluded unless a reliable translated version was accessible.

After duplicate removal, study selection was performed independently by three reviewers (JG, MDB, DLL) in two stages. Titles and abstracts were initially screened to identify studies that potentially met inclusion criteria. Full-text articles were retrieved for studies that satisfied these criteria or in cases of uncertainty based on abstract review. Disagreements regarding study selection were resolved through discussion or, when needed, consultation with a senior author (MK). Reasons for exclusion at the full-text stage were documented. Inter-reviewer agreement was quantified using Cohen's kappa (κ) statistic and interpreted according to standard thresholds: slight (0.00–0.21), fair (0.21–0.40), moderate (0.41–0.60), substantial (0.61–0.80), and almost perfect agreement (0.81–1.00).^11^

### Data extraction

Data extraction was conducted independently by two reviewers (JG, PV) using a standardized data extraction form on Microsoft Excel (version 16.90). Extracted variables included study characteristics (author, year, country, study design, level of evidence, and sample size), patient demographics (mean age, sex distribution, laterality), tear characteristics (definition of irreparability, extent of tear), surgical details (type of tendon transfer) rehabilitation protocols (where reported), and clinical outcome measures. When available, outcomes of interest included validated functional scores, range of motion assessments, pain scores, complication rates, reoperation or revision surgery rates, and structural integrity assessments.

### Quality assessment

The methodological quality of included studies was assessed independently by two reviewers (CK, PV) using the Methodological Index for Non-Randomized Studies (MINORS) criteria. For non-comparative studies, a maximum score of 16 was possible, while comparative studies were scored out of 24.^12^ Each item on the MINORS checklist was rated as 0 (not reported), 1 (reported but inadequate), or 2 (reported and adequate). Discrepancies in scoring were resolved by consensus with input from a senior author (MK). For this review, non-comparative studies were classified as poor (≤8), moderate (9–14), or good quality (15–16), while comparative studies were classified as poor (≤14), moderate (15–22), or good quality (23–24).^12^

### Data synthesis

Due to heterogeneity in study designs, surgical techniques, and reported outcome measures, a quantitative meta-analysis was not feasible. Instead, a narrative synthesis was performed. Descriptive statistics were used to summarize study characteristics and clinical outcomes, with findings organized according to surgical technique where applicable. Clinical outcomes were reported with emphasis on functional improvement, pain reduction, patient-reported measures, complication rates, and graft integrity when available. Data were summarized using absolute frequencies with corresponding percentages or weighted means with measures of variability (weighted standard deviation or range), as appropriate. These weighted means were author-calculated from the raw values available in the studies extracted and organized by treatment type. All analyses were conducted using Microsoft Excel (version 16.90).

## Results

### Search results

**Figure 1.**
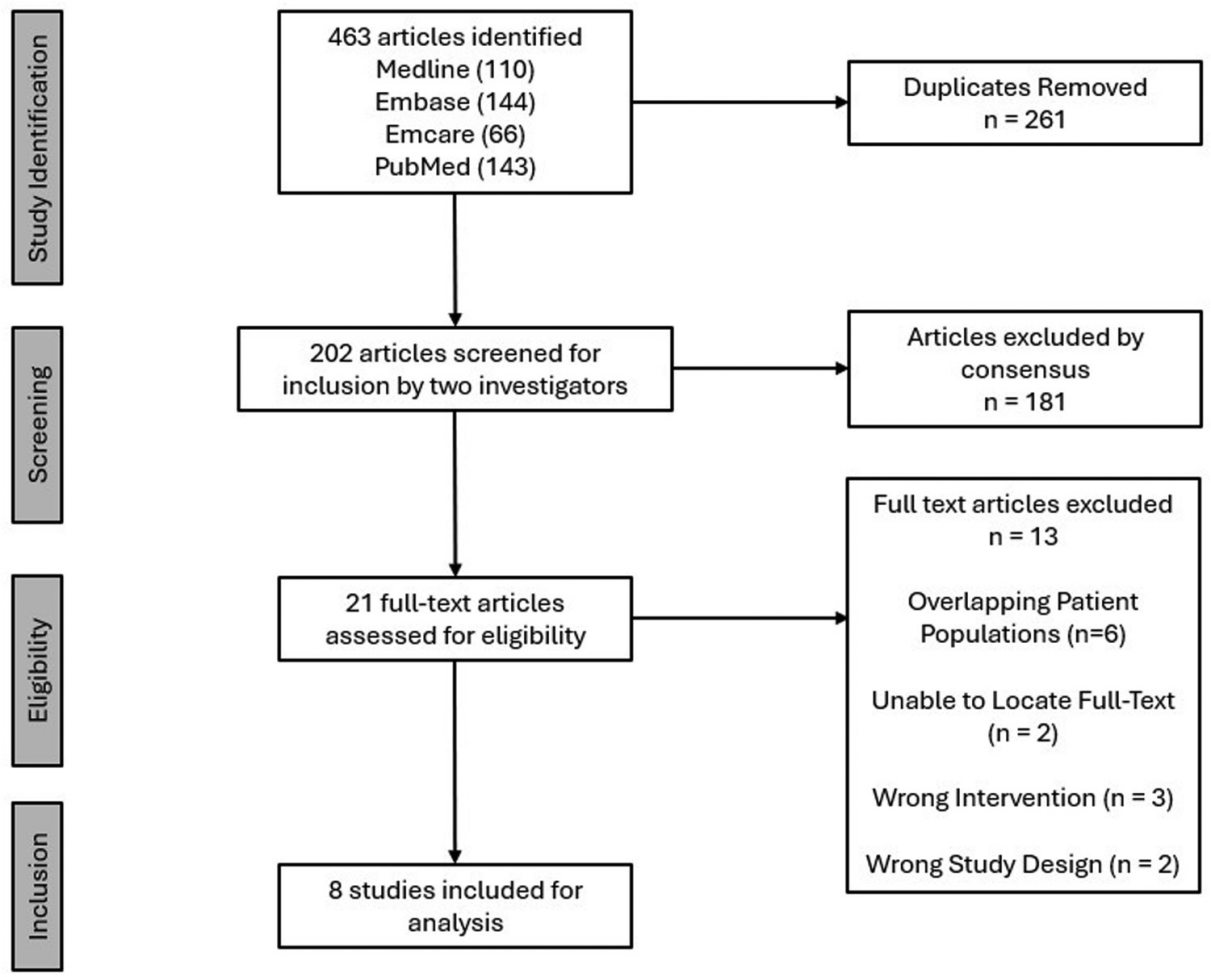
PRISMA diagram for screening process.

A systematic search across **four** electronic databases identified **463** potentially relevant articles. After excluding **261** duplicate articles, **202** articles were available for screening. Agreement between the reviewers was strong at the title and abstract stage (κ = 0.77) and almost perfect at the full-text stage (κ = 0.88). Title and abstract screening excluded **181** articles. After the assessment of **21** full-text articles for eligibility, eight studies satisfied the inclusion criteria and were included in the analysis.^13–20^ Three studies focused on PM transfer,^15–17^ two on Pm tendon transfer,^18,20^ two on LD transfer,^14,19^ and one on combined LDTM transfer^13^ (Figure 1).

**Figure 2.**
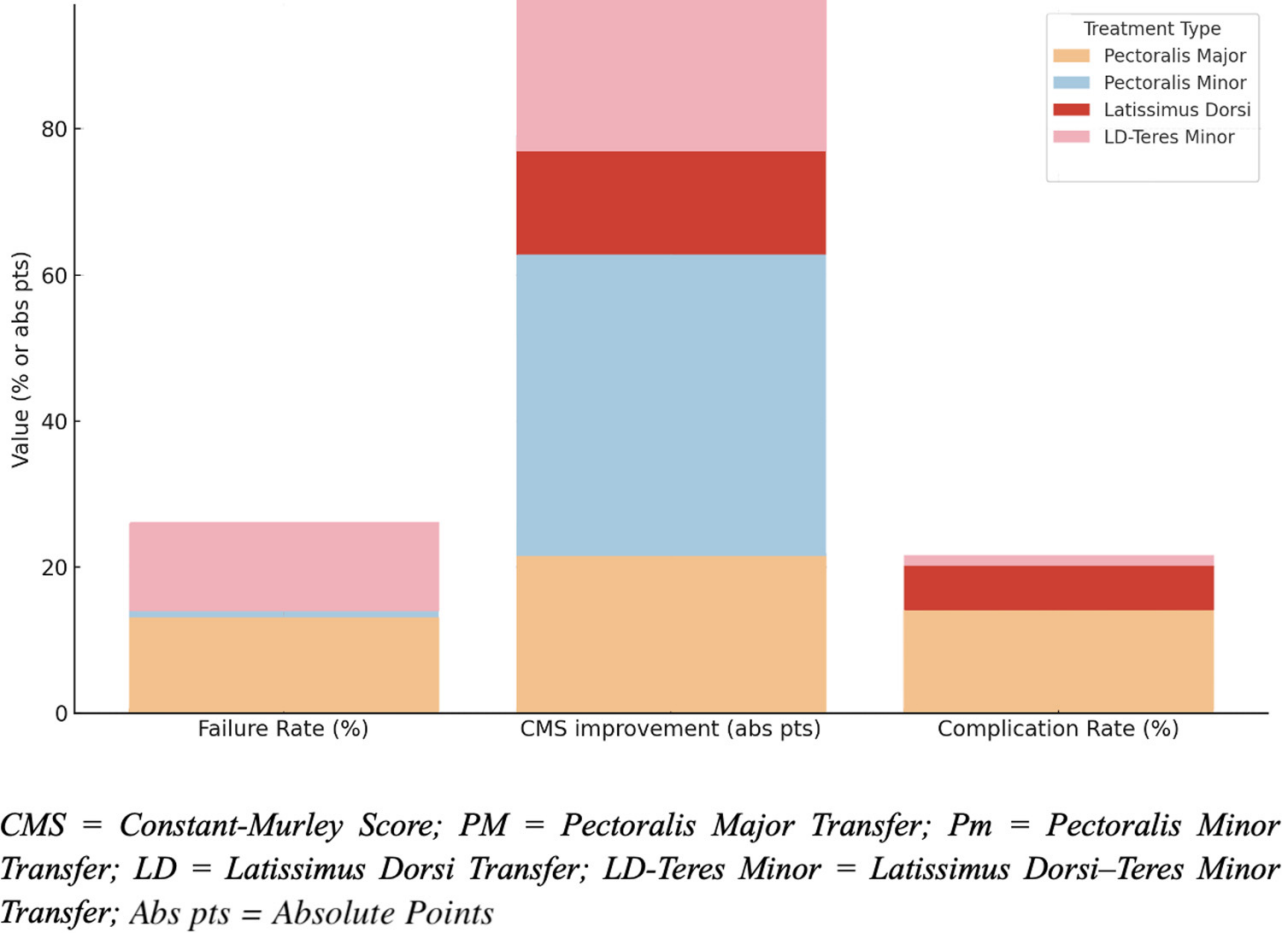
Comparison of failure rates, CMS improvement, and complication rates across reconstruction techniques.

### Study characteristics

Table 1 summarizes the characteristics of the included studies and patient demographics. This review comprised eight studies, encompassing a total of 350 patients (78.0% male, SD = 9.7). The mean age at the time of surgery was 64.8 years (SD = 3.5), with a total mean follow-up duration of 36.9 months (SD = 27.9). The most common study type was retrospective case series (RCS) (*n* = 4),^15,17,18,20^ followed by one retrospective cohort studies,^14^ one retrospective comparative case series,^13^ one prospective clinical trial,^19^ and one retrospective case control study.^16^ The level of evidence ranged from II to IV, with four level IV^15,17,18,20^ studies, three level III^13,14,16^ and one level II.^19^

**Table 1.** Demographics table describing patient characteristics of the eight included studies.

Author (years)	Study design	Level of evidence	Patients (*n*)	Shoulders (*n*)	Mean age (SD)	Male (%)	Mean follow-up, months (SD)	Tendon type
Baek (2024)-1^13^	RCCS	III	123	123	NR	NR	NR	LDTM
Baek (2024)-2^14^	RC	III	23	23	63.7 (5.1)	13/23 (56.5)	24.0 (NR)	LD
Gavriilidis (2010)^15^	RCS	IV	15	15	61.9 (6.6)	10/15 (66.7)	37.0 (NR)	PM
Lederer (2011)^16^	RCC	III	53	54	63.0 (7.3)	40/53 (75.5)	35.0 (14.7)	PM
Moroder (2017)^17^	RCS	IV	22	22	62.4 (8.6)	17/22 (77.3)	120.0 (6.4)	PM
Paladini (2013)^18^	RCS	IV	27	27	60.1 (5.8)	22/27(81.5)	31.0 (4.2)	Pm
Saremi (2023)^19^	Prospective Clinical Trial	II	13	13	NR	NR	12.0 (NR)	LD
Yamakado (2024)^20^	RCS	IV	74	74	69.4 (5.5)	65/74 (87.8)	24.0 (NR)	Pm
Totals, Weighted Means/SD	—	—	**350**	**351**	**64.8 (3.5)**	**78.0 (9.7)**	**36.9 (27.9)**	—

RCCS: retrospective comparative case series; RC: retrospective cohort study; RCS: retrospective case series; RCC: retrospective case control study; NR: not reported; LDTM: latissimus dorsi-teres minor; LD: latissimus dorsi; PM: pectoralis major; Pm: pectoralis minor.

Table 2 presents patient demographics based on treatment type. PM tendon transfer was the most frequently studied procedure, evaluated in three studies (*n* = 90 patients). Though important to note, four of the 53 patients were treated for isolated subscapularis tears rather than AS tears. Pm (*n* = 101) and LD (*n* = 36) transfers were each assessed in two studies (*n* = 101), while combined LDTM transfers (*n* = 123) had one study.

**Table 2.** Patient demographics by treatment type.

Treatment	Number of studies	Sample size	Mean age (SD)	Male % (SD)	Mean follow-Up, months (SD)
Overall	8	350	64.8 (3.5)	78.0 (9.7)	36.9 (27.9)
PM	3	90	62.7 (0.4)	74.5 (5.4)	56.1 (36.3)
Pm	2	101	66.9 (4.1)	86.1 (2.8)	25.9 (3.1)
LD	2	36	63.7 (NA)	56.5 (NA)	19.7 (5.8)
LDTM	1	123	NR	NR	NR

LD: latissimus dorsi; PM: pectoralis major; Pm: pectoralis minor; LDTM: latissimus dorsi teres minor; NA: not applicable; NR: not reported.

The mean age of patients varied slightly by tendon type, with PM and LD transfer patients having the youngest mean ages (62.7 ± 0.4 years and 63.7 years, respectively). Pm had the second-highest mean age at 66.9 ± 4.1 years. LDTM did not have reported ages.

The proportion of male patients also differed across groups, with Pm transfers having the highest percentage of male patients (86.1%). LD and PM transfers had male proportions of 56.5% and 74.5%, respectively.

Follow-up duration also showed some variability, ranging from 19.7 ± 5.8 months for LD to 56.1 ± 36.3 months for PM transfers. Pm transfer follow-up was moderate at 25.9 ± 3.1 months. The sole study on combined LDTM transfers contributed the largest proportion of patients (*n* = 123) yet did not have any reported demographics data for this patient population.

### Study quality

Results of the quality assessment are presented in Supplementary Table 2. Of the eight studies, four are comparative,^13,14,16,19^ and the other four are non-comparative.^15,17,18,20^ For comparative studies, all scored in the moderate methodological quality range from 18 to 21. Similarly, all non-comparative studies fell in the moderate quality range with scores ranging from 10 to 12. These limitations in quality stem primarily from the lack of prospective data collection, prospective sample size calculation, and inability to blind participants and researchers to the surgical techniques used.

### Failure rate

Table 3 contains the failure rates for the various treatment approaches. Treatment failure was defined as a re-tear of the transferred tendon or progression to rTSA. LD transfers had the lowest failure rate at 0%, with no patients in the single LD transfer study experiencing retears or requiring conversion to rTSA. The pooled failure rate for the two Pm transfer studies was 0.99%, as only one out of 101 patients required progression to rTSA. PM and LDTM transfers showed similar failure rates of approximately 12.2%. PM transfer failures ranged from 6.7% to 14.3%, with 10 graft retears and one conversion to rTSA among 90 patients. The single LDTM study reported 10 graft retears and five progressions to rTSA out of 123 patients.

**Table 3.** Total failure rates by treatment type.

Author	Graft retear (*N*)	Conversion to rTSA (*N*)	Total failure rate (*N*)	Total failure rate (%)
Pectoralis minor transfers				
Paladini (2013)^18^	0/27	0/27	0/27	0%
Yamakado (2024)^20^	0/74	1/74	1/74	1.4%
Total Pm	0/101	1/101	1/101	1.0%
Pectoralis major transfers				
Gavriilidis (2010)^15^	1/15	0/15	1/15	6.7%
Lederer (2011)^16^	7/53	0/53	7/53	14.3%
Moroder (2017)^17^	2/22	1/22	3/22	13.6%
Total PM	10/90	1/90	11/90	12.2%
Latissimus dorsi – teres minor transfers				
Baek (2024)-1^13^	10/123	5/123	15/123	12.2%
Latissimus dorsi transfers				
Baek (2024)-2^14^	0/23	0/23	0/23	0%

### PROMs

Table 4 summarizes the patient-reported outcome measures (PROMs) for the different studies and surgical approaches. Measures assessed included the Visual Analog Scale for pain (VAS), American Shoulder and Elbow Surgeons (ASES) score, University of California-Los Angeles (UCLA) Shoulder Scale, Constant-Murley Shoulder Score (CMS), Simple Shoulder Test (SST) score, and the Single Assessment Numeric Evaluation (SANE) score.

**Table 4.** Pre- and postoperative patient-reported outcome measures by treatment type.

Author (year)	Treatment	Patients (*n*)	VAS	ASES	UCLA	CMS	SST	SANE
Gavriilidis (2010)^15^	PM	15	NR	NR	NR	Change: 16.4 Weighted: 246.6	NR	NR
Lederer (2011)^16^	PM	53	NA	NR	NR	Change: 24.6 Weighted: 1303.8	Change: 3.0 Weighted: 159.0	NR
Moroder (2017)^17^	PM	22	NR	NR	NR	Change: 17.6 Weighted: 387.2	Change: 2.0 Weighted: 44.0	NR
Total PM	**3**	**90**	—	—	—	**21.5 (3.7)**	**2.7 (0.5)**	—
Paladini (2013)^18^	Pm	27	Change: −2.2 Weighted: −59.4	NR	NR	Change: 41.2 Weighted: 1112.4	Change: 5.0 Weighted: 135.0	NR
Yamakado (2024)^20^	Pm	74	Change: −5.1 Weighted: −377.4	NR	Change: 15.6 Weighted: 1154.4	NR	NR	NR
Total Pm	**2**	**101**	**−4.3 (1.3)**	—	**15.6 (NA)**	**41.2 (NA)**	**5.0 (NA)**	—
Baek (2024)-2^14^	LD	23	Change: −3.3 Weighted: −75.9	Change: 27.8 Weighted: 639.4	Change: 8.2 Weighted: 188.6	Change: 20.1 Weighted: 462.3	NR	NR
Saremi (2023)^19^	LD	13	Change: −3.5 Weighted: −45.5	NR	NR	Change: 9.2 Weighted: 119.6	NR	NR
Total LD	**2**	**36**	−**3.4 (0.1)**	**27.8 (NA)**	**8.2 (NA)**	**16.2 (5.3)**	—	—
Baek (2024)-1^13^	LDTM	123	Change: −3.1 Weighted: −381.3	Change: 24.8 Weighted: 3050.4	NR	NR	NR	Change: 24.2 Weighted: 2976.6
Total LDTM	**1**	**123**	−**3.1 (NA)**	**24.8 (NA)**				**24.2 (NA)**

PM: pectoralis major; Pm: pectoralis minor; LD: latissimus dorsi; LDTM: latissimus dorsi teres minor; NR: not reported; NA: not applicable.

In the largest cohort (*n* = 123),^13^ LDTM transfers showed improvement in pain and function. VAS scores improved by a mean of 3.1 points, ASES improved by 24.8 points, and CMS rose by 24.2 points from baseline. Two studies (*n* = 36)^14,19^ reported on LD transfer outcomes. The weighted mean improvement in VAS was 3.4 points, ASES improved by 27.8 points, UCLA by 8.2 points, and CMS by 16.2 points. Three studies (*n* = 90)^15–17^ evaluating PM transfers showed a weighted mean CMS improvement of 21.5 points and SST improvement of 2.7 points. Although ASES and VAS were not consistently reported. In 101 patients across two studies^18,20^ evaluating Pm transfers, VAS improved by a weighted mean of 4.3 points. ASES scores improved by 15.6 points, CMS by 41.2 points, and SST by 5.0 points.

### Range of motion

Table 5 summarizes reported preoperative and postoperative shoulder range-of-motion outcomes for the included studies and surgical techniques. Outcomes assessed included forward flexion, abduction, external rotation, and internal rotation, with internal rotation reported either as angular measurement in degrees or as a functional hand-behind-back score based on vertebral level.

**Table 5.** Pre- and postoperative range of motion by treatment type.

Author	Number of patients	FF pre → post (Δ)	ABD pre → post (Δ)	ER pre → post (Δ)	IR (method; Δ)
Pectoralis major transfer					
Gavriilidis 2010	15	145 → 149 (+4)	127 → 135 (+9)	NR	Degrees (+1.3°)
Pectoralis minor transfer					
Paladini 2013	27	127 → 177 (+50)	87 → 163 (+76)	56 → 45 (−11)	Functional (+4.0)
Yamakado 2024	74	104 → 148 (+44)	NR	47 → 57 (+10)	NR
Latissimus dorsi transfer					
Baek 2024-2	23	124 → 155 (+31)	93 → 130 (+37)	51 → 57 (+6)	Functional (+2.7)
Saremi 2023	13	101 → 122 (+21)	111 → 127 (+16)	55.7 → 56.1 (+0.4)	NR
Latissimus dorsi – teres major transfer					
Baek 2024-1	123	104 → 147 (+43)	84 → 123 (+39)	47 → 47 (0)	Functional (+2.5)

FF: forward flexion; ABD: abduction; ER: external rotation; IR: internal rotation; NR: not reported.

In the largest cohort (*n* = 123), Baek et al. reported outcomes following combined latissimus dorsi–teres major transfer, demonstrating substantial improvements in shoulder elevation. Forward flexion improved by 43°, and abduction improved by 39° from baseline, while external rotation showed no net change. Functional internal rotation improved modestly, with a mean increase of 2.5 points on a hand-behind-back scoring scale. Two studies (*n* = 36) evaluating isolated latissimus dorsi transfer reported consistent improvements in elevation. Forward flexion increased by approximately 21–31°, and abduction improved by 16–37° from baseline. External rotation demonstrated minimal improvement, ranging from 0.4° to 6°, while functional internal rotation improved by approximately 2.7 points where reported. One study (*n* = 15) reporting on pectoralis major transfer demonstrated modest improvements in elevation, with forward flexion increasing by 4° and abduction by 9° from baseline. Angular internal rotation improved minimally by 1.3°, while external rotation outcomes were not consistently reported. Two studies (*n* = 101) evaluating pectoralis minor transfer demonstrated the largest reported improvements in shoulder elevation. Forward flexion improved by approximately 44–50°, and abduction improved by up to 76° where reported. External rotation outcomes were variable, with one study reporting a decrease of 11° and another demonstrating an improvement of 10°. Functional internal rotation improved by 4.0 points in one study, while internal rotation was not consistently reported across cohorts.

### Postoperative complications

Table 6 reports the varying complication rates across surgical techniques. Overall, PM transfers demonstrated the highest complication rate at 13.5% (5 of 37 patients),^15,17^ including one infection, two nerve injuries, and two hematomas. The study with the largest cohort of PM transfer patients (*n* = 53)^16^ did not report complications. In contrast, Pm transfers reported no complications among 101 patients.^18,20^ LD transfers had a complication rate of 5.6%, with one infection and one nerve injury reported in a cohort of 36 patients.^14,19^ LDTM transfers demonstrated the lowest overall complication rate among tendon transfers, at 1.6% (2 of 123 patients), with both cases involving infection.^13^ No nerve injuries or hematomas were reported in the LDTM group. A visual comparison of failure rates, Constant-Murley Score (CMS) improvement, and complication rates across tendon transfer techniques is presented in Figure 2.

**Table 6.** Complications by treatment type.

Complications	Pectoralis major (*n* = 37)	Pectoralis minor transfers (*n* = 101)	Latissimus dorsi transfers (*n* = 36)	Latissimus dorsi – teres minor transfers (*n* = 123)
Infection	1	0	1	2
Nerve injury	2	0	1	0
Hematoma	2	0	0	0
Total	5 (13.5%)	0 (0%)	2 (5.6%)	2 (1.6%)

## Discussion

### Summary of key findings

This systematic review evaluated the outcomes of various surgical reconstruction techniques for irreparable AS rotator cuff tears in eight studies comprising 350 patients. Across the included studies, all surgical procedures led to improvements in PROMs, although the magnitude and associated safety profiles varied. Notably, Pm transfers showed the greatest improvement in CMS and VAS pain scores among all tendon transfers. Pm and LD transfers demonstrated the most favorable failure and complication profiles, whereas pectoralis major PM and LDTM transfers had comparatively higher failure rates (12.2%). In terms of objective shoulder motion, tendon transfer procedures were consistently associated with improvements in shoulder elevation, with forward flexion and abduction demonstrating the largest and most reliable gains across techniques. Consistent with PROM improvements, pectoralis minor and latissimus dorsi–based transfers showed the greatest improvements in shoulder elevation, whereas pectoralis major transfer resulted in more modest gains. In contrast, improvements in rotational motion were smaller and more variable, particularly for external rotation. Internal rotation outcomes demonstrated modest functional improvement when reported, although measurement heterogeneity limited direct comparison across studies. Regarding demographics, the age range of patients was narrow among the tendon transfers, ranging from 63.7 to 66.9 years old, however, mean follow-up durations widely varied from 19.7 to 56.1 months.

### Relation to previous work

Prior reviews have examined massive or irreparable cuff tears broadly, without stratifying by anatomical subtypes. This review isolates AS tears, which pose unique biomechanical challenges due to subscapularis involvement, a key stabilizer and internal rotator of the shoulder. The favorable performance of Pm and LD transfers aligns with emerging evidence suggesting biomechanical compatibility with subscapularis function, particularly due to their force vector alignment and contribution to anterior shoulder stability.^21,22^ The observed range-of-motion outcomes provide important biomechanical context to these findings. Across studies, improvements were most pronounced in shoulder elevation, suggesting restoration of anterior force coupling and improved deltoid efficiency rather than true recovery of subscapularis-driven rotational strength. Despite the theoretical advantage of certain transfers in replicating subscapularis anatomy, gains in internal rotation were generally modest and variably reported, highlighting the difficulty of restoring native subscapularis function through tendon transfer alone. However, this contrasts with prior literature, which shows that PM transfers have been favored for AS tears due to its ability to replicate the anatomy of the subscapularis. The lower benefits derived from PM transfers compared to Pm and LD transfers found in this review seems to contradict these prior studies and the sentiment that PM transfers are the gold standard for AS tears.^8,9^ Similarly, LD transfers have been the common choice for irreparable posterosuperior rotator cuff tears, yet this review reveals its potential application for AS tears as well.^23^

Interestingly, while LDTM transfers are theorized to improve scapulohumeral mechanics and strength by combining two muscle units, this review found a relatively high failure rate in the single included study, contrasting with some biomechanical models.^24^ This discrepancy may reflect patient selection or limited data given the sample size. This is to be expected as LDTM transfers are a new construct for AS tears, though the initial promising results warrant further investigations into its potential application.

### Clinical implications

The findings of this review suggest that Pm and LD tendon transfers may offer the most favorable balance of efficacy and safety in managing irreparable AS tears, particularly in younger, active individuals where joint-preserving options are preferred. Pm transfers demonstrated robust functional improvements with no reported complications across a sample of 101 patients, supporting its growing appeal as a viable reconstructive option.^21^

From a functional standpoint, the consistent improvement in shoulder elevation across tendon transfers suggests that these procedures are effective at restoring overhead and forward-reaching activities, which likely contribute substantially to patient-reported functional gains. However, the more limited and inconsistent improvements in rotational motion, particularly internal rotation, should be considered when counselling patients regarding expected postoperative capabilities, especially for tasks requiring behind-the-back or forceful internal rotation. These findings underscore the importance of aligning surgical goals with patient-specific functional demands.

From a surgical planning perspective, this review reinforces the need to weigh biomechanical demands and patient-specific anatomy against potential complications and failure risks. PM and LDTM transfers, while offering meaningful functional gains, appear to carry a greater risk of complications and failure. Despite PM transfers historically being the preferred transfer for irreparable AS tears, this review may suggest its role as the standard could be questioned in favor of more durable and impactful options. For researchers, these results underscore the necessity for high-quality comparative studies of tendon transfers in irreparable AS tears.

### Strengths and limitations

A key strength of this review is its focused evaluation of AS rotator cuff tears, contributing to the limited prior literature that did not account for the anatomical and functional specificity of concurrent subscapularis involvement. Whereas most existing literature on irreparable tears centers on isolated subscapularis or posterosuperior involvement, this study helps to confirm and address a notable gap in current evidence.^4,5^ Additionally, the inclusion of underrepresented techniques such as Pm, LD, and LDTM transfers adds relevance to evolving clinical practice.

However, the quality of evidence was limited. All included studies were non-randomized, with most being retrospective in nature and all being of moderate quality according to the MINORS Criteria. Heterogeneity in surgical techniques, follow-up durations, and outcome reporting precluded meta-analysis. The set of PROMs assessed varied across studies, limiting direct comparison of functional outcomes between transfers. Furthermore, complication reporting was inconsistent, with several studies omitting definitions or not reporting complications at all, particularly in larger series. The study contributing the largest number of patients treated with PM transfers did not report any complication data.^16^ These limitations hindered clear comparisons of safety and efficacy across techniques.

Interpretation of range-of-motion outcomes was limited by heterogeneous reporting across studies. Internal rotation was variably assessed using angular measurements or functional hand-behind-back scoring systems, precluding quantitative synthesis. Additionally, inconsistencies in follow-up duration and reporting of standard deviations limited the ability to pool ROM outcomes or perform comparative statistical analyses. As such, ROM findings were summarized descriptively and interpreted in conjunction with patient-reported outcomes.

The relative paucity of data on techniques, often limited to single studies or small sample sizes further restricts generalizability and limits the ability to form robust recommendations. For instance, while 123 patients were assessed for LDTM transfers, this came from a single study, and though three studies reported on PM transfers, only 90 patients were included. Future studies with larger cohorts, standardized PROMs, clear complication definitions, and consistent follow-up are needed to allow for meaningful comparisons and better inform surgical decision-making in this complex patient population.

## Conclusion

Despite several proposed surgical strategies, there remains no clear consensus on the optimal management of irreparable AS rotator cuff tears. This review found that all techniques demonstrated postoperative improvements in clinical outcomes with pectoralis minor transfers having notably favorable efficacy and safety profiles, though these results should be interpreted with caution. Comparisons and analysis were limited by heterogeneity in study design, outcome measures, and follow-up. The existing evidence is largely derived from retrospective, non-randomized studies of moderate methodological quality. These limitations underscore the need for high-quality, standardized, and prospective comparative trials to better define the safety and efficacy of reconstructive strategies in this complex patient population.

## Supplemental Material

sj-docx-1-sel-10.1177_17585732261431826 - Supplemental material for Soft tissue reconstruction techniques for irreparable anterosuperior rotator cuff tears: A systematic review of clinical outcomes Supplemental material, sj-docx-1-sel-10.1177_17585732261431826 for Soft tissue reconstruction techniques for irreparable anterosuperior rotator cuff tears: A systematic review of clinical outcomes by Justin Gilbert, Marc Daniel Bouchard, Colin Kruse, Prushoth Vivekanantha, Darius Luke Lameire, Danielle Dagher and Moin Khan in Shoulder & Elbow

sj-docx-2-sel-10.1177_17585732261431826 - Supplemental material for Soft tissue reconstruction techniques for irreparable anterosuperior rotator cuff tears: A systematic review of clinical outcomes Supplemental material, sj-docx-2-sel-10.1177_17585732261431826 for Soft tissue reconstruction techniques for irreparable anterosuperior rotator cuff tears: A systematic review of clinical outcomes by Justin Gilbert, Marc Daniel Bouchard, Colin Kruse, Prushoth Vivekanantha, Darius Luke Lameire, Danielle Dagher and Moin Khan in Shoulder & Elbow
