# Supplementary material for: Soft tissue reconstruction techniques for irreparable anterosuperior rotator cuff tears: A systematic review of clinical outcomes
Source: Shoulder Elbow. 2026 Mar 18:17585732261431826. Online ahead of print. doi: 10.1177/17585732261431826 (PMC12999534; doi:10.1177/17585732261431826)
Supplement: sj-docx-1-sel-10.1177_17585732261431826 - Supplemental material for Soft tissue reconstruction techniques for irreparable anterosuperior rotator cuff tears: A systematic review of clinical outcomes [file sj-docx-1-sel-10.1177_17585732261431826.docx]

**Supplementary Table 1.** Electronic search strategies used for Ovid MEDLINE, Embase, Emcare, and PubMed databases.

**Ovid MEDLINE(R) ALL <1946 to April 6, 2024>**

1. Anterior (481536)
2. Superior (474436)
3. 1 and 2 (28715)
4. Anterosuperior (1449)
5. 3 or 4 (29990)
6. Exp Rotator cuff or “rotator cuff”.mp or shoulder.mp (109050)
7. 5 and 6 (2284)
8. Tear or exp Tears/ (49842)
9. Rupture or exp Rupture/ (166097)
10. Instab* or unstab* (275942)
11. injur* (1520495)
12. 8 or 9 or 10 or 11 (1905591)
13. Irreparable or Unfixable or Irrecoverable or Irreversible (92186)
14. Unrepairable or Incurable or Unmendable (16740)
15. 13 or 14 (108745)
16. 7 and 12 and 15 (110)

**Embase <1974 to 2025 April 6>**

1. Anterior (663701)
2. Superior (628360)
3. 1 and 2 (45594)
4. Anterosuperior (1850)
5. 3 or 4 (47198)
6. “Rotator cuff.mp” exp Rotator Cuff/ or shoulder.mp (143175)
7. 5 and 6 (2886)
8. Tear (73201)
9. Rupture or exp Rupture/ (307793)
10. Instab* or unstab* (376786)
11. injur* (2145563)
12. 8 or 9 or 10 or 11 (2751084)
13. Irreparable or Unfixable or Irrecoverable or Irreversible (115913)
14. Unrepairable or Incurable or Unmendable (26862)
15. 13 or 14 (142500)
16. 7 and 12 and 15 (144)

**Ovid Emcare <1995 to 2025 Week 15>**

1. Anterior (166017)
2. Superior (135287)
3. 1 and 2 (12199)
4. Anterosuperior (758)
5. 3 or 4 (12857)
6. “Rotator cuff.mp” exp Rotator Cuff/ or shoulder.mp (62391)
7. 5 and 6 (1418)
8. Tear (20735)
9. Rupture or exp Rupture/ (90516)
10. Instab* or unstab* (91163)
11. injur* (589367)
12. 8 or 9 or 10 or 11 (734034)
13. Irreparable or Unfixable or Irrecoverable or Irreversible (16479)
14. Unrepairable or Incurable or Unmendable (4710)
15. 13 or 14 (21133)
16. 7 and 12 and 15 (66)

**PubMed <inception to January 29, 2026>**

1. anterior[tiab]
2. superior[tiab]
3. 1 AND 2
4. anterosuperior[tiab]
5. 3 OR 4
6. "Rotator Cuff"[Mesh] OR "Rotator Cuff Injuries"[Mesh] OR rotator cuff[tiab] OR shoulder[tiab]
7. 5 AND 6
8. tear*[tiab]
9. rupture*[tiab]
10. instab*[tiab] OR unstab*[tiab]
11. injur*[tiab]
12. 8 OR 9 OR 10 OR 11
13. irreparable[tiab] OR unfixable[tiab] OR irrecoverable[tiab] OR irreversible[tiab]
14. unrepairable[tiab] OR incurable[tiab] OR unmendable[tiab]
15. 13 OR 14
16. 7 AND 12 AND 15
