# Supplementary material for: Soft tissue reconstruction techniques for irreparable anterosuperior rotator cuff tears: A systematic review of clinical outcomes
Source: Shoulder Elbow. 2026 Mar 18:17585732261431826. Online ahead of print. doi: 10.1177/17585732261431826 (PMC12999534; doi:10.1177/17585732261431826)
Supplement: sj-docx-2-sel-10.1177_17585732261431826 - Supplemental material for Soft tissue reconstruction techniques for irreparable anterosuperior rotator cuff tears: A systematic review of clinical outcomes [file sj-docx-2-sel-10.1177_17585732261431826.docx]

**Supplementary Table 2.** Methodological quality assessment of included studies using the Methodological Index for Non-Randomized Studies (MINORS) criteria.

|  | *^Baek 2024-1^* | *^Baek 2024-2^* | *^Gavriilidis 2010^* | *^Lederer^*  *^2011^* | *^Moroder^*  *^2017^* | *^Paladini^*  *^2013^* | *^Saremi^*  *^2023^* | *^Yamakado^*  *^2024^* |
| --- | --- | --- | --- | --- | --- | --- | --- | --- |
| *1. A clearly stated aim* | 2 | 2 | 2 | 2 | 2 | 2 | 2 | 2 |
| *2. Inclusion of consecutive patients* | 1 | 1 | 1 | 2 | 2 | 2 | 1 | 2 |
| *3. Prospective collection of data* | 1 | 1 | 0 | 1 | 1 | 1 | 2 | 1 |
| *4. Endpoints appropriate to the aim of study* | 2 | 2 | 2 | 2 | 2 | 2 | 2 | 2 |
| *5.Unbiased assessment of study endpoint* | 1 | 1 | 1 | 2 | 1 | 0 | 1 | 1 |
| *6. Follow-up period appropriate for to the aim of study* | 2 | 2 | 2 | 2 | 2 | 2 | 1 | 2 |
| *7. Loss to follow-up less than 5%* | 2 | 2 | 2 | 2 | 1 | 2 | 2 | 2 |
| *8. Prospective calculation of study size* | 0 | 2 | 0 | 0 | 0 | 0 | 0 | 0 |
|  |  |  |  |  |  |  |  |  |
| *9. An adequate control group* | 2 | 2 |  | 2 |  |  | 2 |  |
| *10. Contemporary groups* | 2 | 1 |  | 2 |  |  | 2 |  |
| *11. Baseline equivalence of groups* | 2 | 2 |  | 2 |  |  | 1 |  |
| *12. Adequate statistical analyses* | 2 | 2 |  | 2 |  |  | 2 |  |
| *TOTAL MINORS SCORE* | **19** | **20** | **10** | **21** | **11** | **11** | **18** | **12** |
| *Maximum possible score* | **24** | **24** | **16** | **24** | **16** | **16** | **24** | **16** |

**Legend (Total MINORS Score)** poor quality; moderate quality; good quality
